# An evaluation of the diagnostic performance characteristics of the Yellow Fever IgM immunochromatographic rapid diagnostic test kit from SD Biosensor in Ghana

**DOI:** 10.1371/journal.pone.0262312

**Published:** 2022-01-07

**Authors:** Lawrence Henry Ofosu-Appiah, Dodzi Kofi Amelor, Bright Ayensu, Ernest Akyereko, Nafisah Issah Rabiwu, David Opare, Godfred Owusu-Okyere, Dennis Odai Laryea, Franklin Asiedu-Bekoe, Julius Abraham Addo Mingle

**Affiliations:** 1 National Public Health and Reference Laboratory, Ghana Health Service, Korle-Bu, Accra, Ghana; 2 Disease Surveillance Department, Ghana Health Service, Korle-Bu, Accra, Ghana; 3 Disease Control Department, Ghana Health Service, Korle-Bu, Accra, Ghana; 4 Department of Medical Microbiology, School of Medicine and Dentistry, College of Health Sciences, University of Ghana, Korle-Bu, Accra, Ghana; Qatar University, QATAR

## Abstract

Yellow fever is endemic in Ghana and outbreaks occur periodically. The prodromal signs due to Yellow Fever Virus (YFV) infection are non-specific, making clinical signs unreliable as the sole criteria for diagnosis. Accurate laboratory confirmation of suspected yellow fever cases is therefore vital in surveillance programs. Reporting of ELISA IgM testing results by laboratories can delay due to late arrival of samples from the collection sites as well as limited availability of ELISA kits. In this study, the diagnostic performance characteristics of a rapid immunochromatographic Standard Q Yellow Fever IgM test kit (SD Biosensor) was evaluated for the rapid diagnosis of Yellow Fever infection in Ghana. A panel of 275 sera, comprising 81 confirmed YFV positives and 194 negatives were re-tested in this study using the Standard Q Yellow Fever IgM test kit. Using the CDC/WHO Yellow Fever IgM capture ELISA as a benchmark, the sensitivity, specificity and accuracy of the Standard Q Yellow Fever test kit were 96.3%, 97.9% and 97.5%, respectively. The false positivity rate was 5.1% and there was no cross-reactivity when the Standard Q Yellow Fever test kit was tested against dengue, malaria and hepatitis B and C positive samples. In addition, inter-reader variability and invalid rate were both zero. The results indicate that the diagnostic performance of the Standard Q Yellow Fever IgM test kit on serum or plasma is comparable to the serum IgM detection by ELISA and can be used as a point of care rapid diagnostic test kit for YFV infection in endemic areas.

## Introduction

Yellow fever is an acute febrile illness caused by the Yellow Fever Virus (YFV). The symptoms of infection generally occur 3–6 days after exposure to the virus. The clinical presentation of infection in humans ranges from mild illness with flu-like symptoms fever, headache, nausea, muscle pain, backache, vomiting, jaundice [1] to severe illness which can occur in 25–50% of cases. Severe illness can progress into full hemorrhagic syndrome with multiorgan failure [2, 3].

The virus belongs to the family *Flaviviridae* and genus *Flavivirus*. It is an enveloped virus and its genome consists of a single-stranded positive sense RNA (approximately 11 kb). The virus is phylogenetically classified into seven genotypes [4]. The *Aedes aegypti* mosquito is the primary vector of the YFV and is geographically restricted to the tropical regions of the world due to its inability to survive in colder climates [5]. As such, YFV contributes to a significantly high disease burden in the tropical regions of Africa and South America where outbreaks occur from time to time [2, 6–8].

Symptoms of YFV infection resemble those of a wide range of diseases including dengue, zika, other hemorrhagic viral diseases, leptospirosis, viral hepatitis, and malaria. This makes clinical signs unreliable as the sole diagnostic criteria of YFV infection. Laboratory confirmation of suspected YFV infection remains critical in the diagnosis of YF [7, 9, 10]. There is no doubt that laboratory confirmation has become the cornerstone of YF case-based surveillance programs.

Laboratory testing for YFV has evolved considerably from traditional methods such as Plaque Reduction Neutralisation Test (PRNT), Haemagglutination Inhibition Assay, Immunofluorescence Assay and Western Blot to more recent platform and serological assays such as Rapid Microneutralisation Assay, Lateral Flow and Microsphere Immunoassay [11]. Serological diagnosis depends on detecting YFV host antibodies in the serum of infected individuals. Neutralisation assays are considered to provide the greatest specificity of all the currently available serological assays. While the PRNT remains the gold standard for the serological diagnosis of YFV infection, it is laborious and takes several days to weeks [12].

The use of Polymerase Chain Reaction (PCR) has made virus detection quite simpler but the primers and probes as well as the needed equipment might not be available in less endowed laboratories. More importantly the persistence of viral RNA in serum is short lived making it difficult to detect in samples collected more than 6 days post onset of symptoms. Laboratory diagnosis of YF currently depends on specific IgM detection by Enzyme Linked Immuno-Sorbent Assay [13]; however, cross-reactivity with other co-circulating flaviviruses can occur due to shared cross-reactive epitopes on the flavivirus E protein [10].

Molecular diagnostic testing is largely unavailable at lower level laboratories and this is coupled with the unavailability of facilities to maintain cold chain needed for storage of reagents for molecular testing [14–17].

Although molecular and ELISA testing systems are available in reference or public health laboratories, the reporting of results from IgM testing can take several days. This delay can be due to slow transportation of clinical specimens from the collection site to the reference laboratory because of remote locations, limited transport infrastructure, and lack of local resources. Frequent limited availability of ELISA kits also leads to delays in testing of samples from suspected cases and reporting of results. The current CDC/WHO IgM capture ELISA uses different components from different sources, which can have negative implications for the sensitivity of the reagents and the overall outcome of the test results. The availability of one component may not be readily complemented by the availability of other components leading to delays in testing of samples from suspected cases.

In the light of the above challenges, it will be ideal to employ simple, easy to use and accurate rapid diagnostic tests as point-of care tests (POCTs) in areas where yellow fever is endemic. However independent validation and implementation of such POCTs remain limited. This study therefore evaluated the diagnostic performance characteristics of the Standard Q Yellow Fever IgM test kit from SD Biosensor Inc., for the rapid diagnosis of YF infection in human serum samples arising from natural infections by comparing its diagnostic performance with the sandwich ELISA protocol developed by CDC/WHO and used by laboratories in the WHO Yellow Fever Laboratory Network including Ghana [18].

## Methods

### Kit to be evaluated

The STANDARD™ Q Yellow Fever IgM Test kit is a rapid diagnostic (RDT) kit from SD Biosensor Inc. (S1 Table). The analyte is IgM antibodies and the sample types this product is suitable for include serum, plasma and whole blood.

The test kit has “T” as test line and “C” as control line. Monoclonal anti-human IgM is immobilized at the test line (T line) on the nitrocellulose membrane. Inactivated Yellow fever virus in the antigen pad and monoclonal anti-Yellow fever-env-gold in the conjugate pad are released by adding assay diluent and react with anti-Yellow Fever IgM in patient sample. If human anti-Yellow Fever IgM exists in the test sample, the individual test line appears as a visible band respectively forming the complex with anti-human IgM, human IgM, inactivated Yellow fever virus, and anti-Yellow fever-env-gold, which indicates a positive test result. The violet line at the control region should always appear if the assay is performed correctly. This kit should be stored at room temperature, 2–40°C / 36–104°F, out of direct sunlight. Kit materials are stable until the expiration date printed on the outer box. At the time of this evaluation, the accuracy of this kit was not known.

### Sample selection and study design

In this study, a total of 275 serum samples were used for the RDT evaluation; 215 from an archived pool of serum samples and a further 60 from an ongoing outbreak. The archived serum samples that were tested by the STANDARD™ Q Yellow Fever IgM test kit were collected from persons across all the regions of Ghana between 2010 and 2018, while the extra 60 samples were collected in 2021 mainly from the northern part of the country. All the samples were collected as part of the national yellow fever surveillance program in Ghana. The samples were transported to the NPHRL in Accra and tested for anti-yellow fever specific IgM antibodies using the sandwich ELISA protocol developed by CDC/WHO [18] prior to archiving at -80°C (S1 Text). These included: 81 samples that tested positive by YFV IgM capture ELISA at the National Public Health Reference Laboratory in Accra, Ghana and confirmed by PRNT [19] and or RT-PCR [16] at the Institute Pasteur in Dakar, Senegal; 194 samples that tested negative by YFV IgM ELISA hence not tested by PRNT or RT-PCR as stipulated in the WHO YF testing protocol (S2 Table).

### Testing of serum samples using the Standard Q Yellow Fever IgM test

The tests were performed according to the manufacturer’s instructions. The test device was placed on a flat surface and 10 ul of serum was added to the sample well. 90 ul of assay diluent were added into the assay diluent well of the test device. The test results were read at 15–20 minutes. To avoid false results, no test result was read after 20 minutes. The temperature of the testing laboratory environment was recorded each day of the evaluation period to ensure that it was not significantly different from the manufacturer’s recommended room temperature of 25°C. A total of 5 room temperature readings with an average of 27.9°C were recorded on 5 different testing days.

### Specificity test using known positive samples from other febrile cases

The specificity of the Yellow Fever IgM rapid diagnostic test kit was also examined by testing its cross-reactivity on samples from patients with other febrile illnesses. These were 17 plasma samples derived from children with *Plasmodium falciparum* infection; 7 sera from hepatitis B surface antigen positive cases; 5 sera from hepatitis C antibody positive cases; and 2 samples that tested positive for Dengue NS1 antigen (S2 Table). These samples had already been tested, passed the retention period and were due to be discarded.

### Quality assurance

One biomedical scientist performed the assay procedure using the kit on any particular day of the evaluation exercise. The result of each test was then independently read visually and interpreted by three biomedical scientists. Two concordant results out of three reading results determined the final outcome.

### Data management and statistical analysis

We obtained data on archived yellow fever samples collected between 2010 to 2018 as well as those collected during the 2021 outbreak. The data was entered into Microsoft Excel version 2016 and exported to STATA Version 15 for analysis. A contingency table for the Standard Q Yellow Fever IgM RDT and YF IgM Capture ELISA was thus created (S3 Table). The Performance of the YF IgM RDT against the YF IgM capture ELISA test was determined by calculating diagnostic sensitivity and specificity, as well as negative and positive predictive values. Diagnostic sensitivity was defined as the ability of the test kit under evaluation to correctly detect samples that contain yellow fever IgM antibodies (positive by sandwich ELISA and RT-PCR). Sensitivity was therefore calculated as the number of true positive samples identified by the YF IgM Test as positive, divided by the number of specimens identified in the serum panel as positive, expressed as a percentage. Diagnostic specificity, defined as the ability of the kit under evaluation to correctly detect specimens that do not contain yellow fever IgM antibodies (negative by sandwich ELISA), was calculated as the number of true negative specimens identified by the test kit under evaluation as negative, divided by the number of specimens identified in the reference panel as negative, and expressed as a percentage. Negative predictive value was calculated as the proportion of those with a negative test result who are uninfected and positive predictive value as the proportion of those with a positive test result who are actually infected [20].

The detection limit of the YF IgM RDT was also determined using pooled positive yellow fever IgM sera serially diluted with yellow fever IgM negative serum. The inter-reader variability was then expressed as the percentage of samples for which initial test results were differently interpreted (either positive or negative) by the independent readers.

## Results

**Fig 1:**
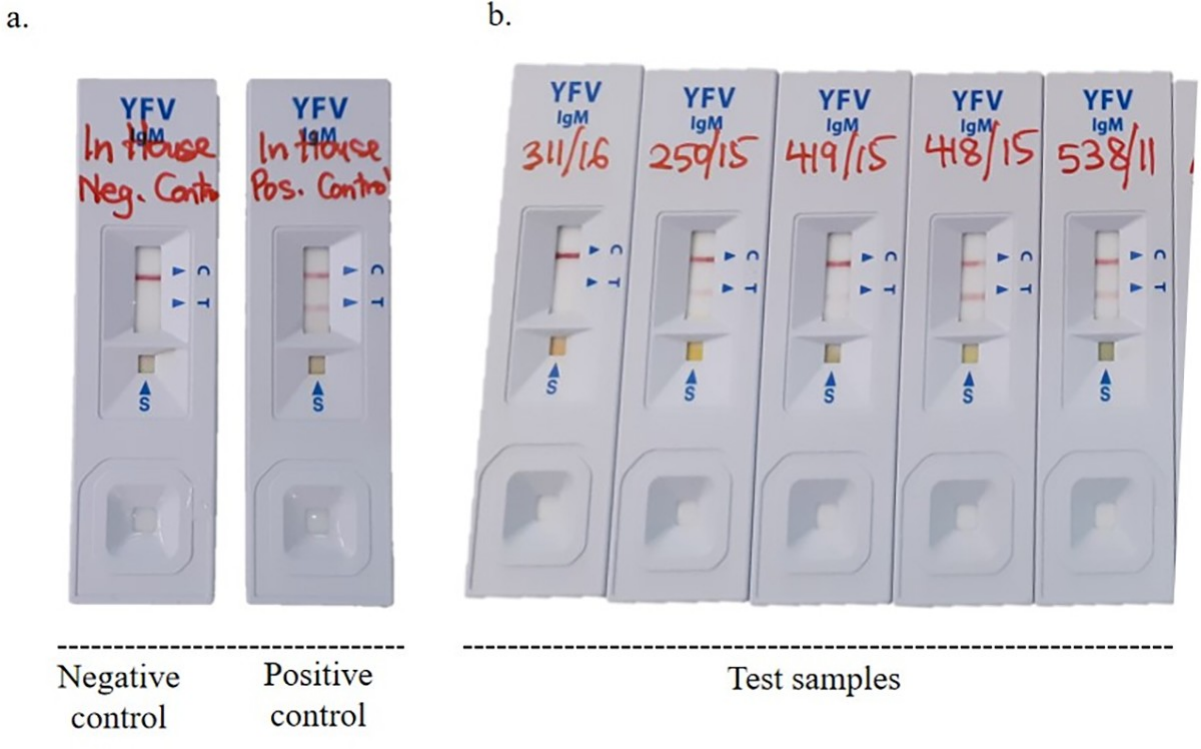
Results of YFV IgM test using STANDARD™ Q Yellow Fever IgM test kit. (a) Represents results of in-house negative and in-house positive controls. The in-house negative control consists of serum negative for YFV IgM antibodies based on ELISA and PRNT results whereas the in-house positive control consists of serum confirmed to contain YFV IgM antibodies based on ELISA and PRNT results. (b) Test results of serum samples collected from persons with suspected YFV. Point S indicates sample well; T indicates test line and C indicates control line.

Specimen processing time for the YF IgM test was found to be minimal and results were available within 20 minutes by interpreting the presence or absence of a band that appears in the test window of the RDT device. Independent reading by the biomedical scientists was shown to be accurate and the test bands could be photographed for records and for real time audit or a second opinion using a mobile phone (Fig 1). The YF IgM Test invalid rate, which is a ratio of number of invalid YF IgM Test results to total number of YF IgM Test results expressed as a percentage, was zero.

### Performance characteristics

The RDT was able to correctly diagnose 96.30% of the samples (sensitivity) and correctly identify 97.94% of non-cases (specificity). The positive predictive value (PPV) of the RDT defined as the probability that when the test is reactive then that specimen actually does contain yellow fever IgM antibodies was 95.12% whereas the negative predictive value (NPV) defined as the probability that when the test is negative then that specimen truly does not contain yellow fever IgM antibodies was 98.44%. The overall level of agreement between the RDT and the ELISA was 97.45% (Tables 1 and 2, S4 and S5 Tables).

**Table 1:** Comparison of the RDT and ELISA results.

N = 275	ELISA IgM Result		
	Negative	Positive	Total
**RDT Final Result**			
Negative	190 (97.94%)	3 (3.70%)	193 (70.19%)
Positive	4 (2.06%)	78 (96.30%)	82 (29.82%)
Total	194 (100.00%)	81 (100.00%)	275 (100.00%)

**Table 2:** Diagnostic performance characteristics of the Standard Q Yellow Fever IgM test kit and level of agreement with YFV IgM ELISA.

Statistic	Formula	Calculation	Result	95% Confidence Interval
Sensitivity	(TP/TP+FN) *100	78/81*100	96.30%	(92%-100%)
Specificity	(TN/TN+FP) *100	190/194*100	97.94%	(66%-100%)
Positive Predictive Value	(TP/TP+FP) *100	78/82*100	95.12%	(94%-100%)
Negative Predictive Value	(TN/TN+FN) *100	190/193*100	98.44%	(60%-98%)
False Omission Rate	FN/TN	3/190	0.0158	
False Discovery Rate	FP/TP	4/78	0.0513	
Level of Agreement	TN+TP/Total	(190+78/275) *100	97.45%	94%-100%

### Specificity test using known positive samples from other febrile cases

Apart from the calculated specificity, there was also no cross-reactivity observed when the YF IgM test was subjected to serum and plasma samples from malaria, and hepatitis B and C antibody positive cases. Similarly, the YF IgM test showed negative when tested with dengue positive samples.

### Detection of YF IgM antibodies with respect to number of days post onset of symptoms

The percentage of positive samples detected by the YFV IgM Test, and IgM ELISA at different times after onset of fever with jaundice is shown in Table 3. The data from the YFV IgM Test were found to be in agreement with those from IgM ELISA of confirmed YF samples.

**Table 3:** Detection of IgM with respect to number of days post onset of symptoms: YFV ELISA vrs Standard Q YF RDT.

Days Post Onset	Total ELISA Positives	Number Positive by YF IgM Test	% Positive by YF IgM Test
Less than 7 days	68	65	96
8 to 14 days	9	9	100
15 to 21 days	2	2	100
22 to 28 days	2	2	100

### Detection limit of YFV IgM test kit

Inter-reader variability using undiluted serum samples was zero. A discordance in interpreting results was observed at a serum dilution of 1 in 20 parts per volume of negative sera. The YFV IgM test kit was observed to show negative results below a serum dilution of 1 in 20 parts per volume of negative sera. The detection limit was subsequently determined to be at a YFV IgM serum concentration of 0.0608 IU/μL (shown in bold italics in Table 4).

**Table 4:** Calculation of detection limit of the YFV IgM test kit.

1 in 20 Serial Dilution					
Sample ID	YF IgM Conc (IU/μL)	RDT Result 1	RDT Result 2	RDT Result 3	Final Result
Negative Control	0.0000	Negative	Negative	Negative	**Negative**
Positive Control	0.6080	Positive	Positive	Positive	**Positive**
Dil 9/11-	0.2736	Positive	Positive	Positive	**Positive**
Dil 8/12-	0.2432	Positive	Positive	Positive	**Positive**
Dil 7/13-	0.2128	Positive	Positive	Positive	**Positive**
Dil 6/14-	0.1824	Positive	Positive	Positive	**Positive**
Dil 5/15-	0.1520	Positive	Positive	Positive	**Positive**
Dil 4/16-	0.1216	Positive	Positive	Positive	**Positive**
Dil 3/17-	0.0912	Positive	Positive	Positive	**Positive**
***Dil 2/18-***	***0*.*0608***	***Positive***	***Positive***	***Negative***	***Positive***
Dil 1/19-	0.0304	Negative	Negative	Negative	**Negative**

## Discussion

Since the early 1920s, yellow fever has been a huge burden in West Africa and Ghana with epidemics occurring from time to time [21–23]. Due to the broad range of clinical symptoms, early and accurate laboratory diagnosis is essential for appropriate patient management [7, 9]. Diagnosis of YFV in the laboratory is contingent on virus or antibody detection in clinical material but timing of sample collection is essential for proper diagnostic interpretation [24]. This is because the cardinal signs and symptoms used in clinical diagnosis appear at different times of infection; during which viral RNA, IgM or IgG antibodies may or may not be present [1, 25]. As with all assays based on antibody detection, the early acute disease period usually presents a negative window of detection, given the need for the relevant antibody response to be elicited [3].

Detection of YFV infection by serology is complicated in areas of the world where other flaviviruses co-circulate (e.g., Dengue, Japanese encephalitis, West Nile and more recently, Zika virus), because of shared cross-reactive epitopes on the flavivirus E protein, and hence cross-reactivity of the antibody response. Antibodies directed against these flaviviruses can cross-react in YFV serology assays, leading to false-positive results [10, 24, 26]. The observed non-cross-reactivity with the 2 dengue positive samples could be due to the fact that these were actually NS1 positive samples with no evidence of dengue IgM and hence the absence of cross-reactivity.

A highly sensitive and yet specific diagnostic test kit becomes useful in producing timely and efficient laboratory diagnosis [27]. This RDT can be employed to yield timely (same day) results which will allow early detection of outbreaks and the institution of appropriate patient management [28], and disease control measures such as vaccination or spraying of vector infested areas.

Detection of YFV specific IgM in the absence of recent YF vaccination and negative diagnosis, including IgM antibodies, for other flaviviruses is considered confirmatory of YF [11]. The persistence of IgM antibodies for longer periods has been reported in a small percentage of vaccinees, which could interfere with diagnostic testing. Also, caution should be exercised in the interpretation of IgM test results possibly with epidemiological considerations and last known date of vaccination since anti-YFV IgM antibodies can be detected up to 3 months after infection [11, 29].

Rapid diagnostic tests or assays whose performance are similar to standard ELISA based tests and which also differentiates yellow fever from other pathogens with similar clinical presentations are expected to exhibit high (analytical and) clinical sensitivity and specificity capable of predicting the true diseased state of an individual in a population of low or high endemicity [30].

The present study places the clinical sensitivity and specificity well above 95%. Diagnostic specificity and Positive Predictive Value of the YF IgM test are not impacted, however, by the test’s inherent non-reactivity against other flavivirues like hepatitis C virus, as shown by the present study. It would be good to subject the YFV IgM test kit to IgM positive samples from other Flaviviruses such as Dengue, Zika and West Nile to determine the extent of non-reactivity since this was not done in the present study.

Usually assays with high clinical sensitivity (i.e., few negative results among patients with high likelihood of disease) may exhibit reduced clinical specificity (i.e., many positive results among patients with low likelihood of disease). Conversely, high specificity may be achieved at the cost of reduced clinical sensitivity. It was interesting to note that the Standard Q yellow fever IgM test did not exhibit such a significant bias.

## Conclusion

Our results indicate that the diagnostic performance of the Standard Q Yellow Fever IgM test kit from SD Biosensor on serum or plasma is comparable to the current serum CDC/WHO YF IgM capture ELISA assay. The kit demonstrated acceptably high diagnostic sensitivity and specificity values for diagnosis of yellow fever infection. This suggests that in countries where yellow fever is endemic, and where access to confirmatory laboratory testing is limited, the Standard Q yellow fever IgM test could serve as an acceptable test and can be used by medical practitioners as a point of care test kit for the rapid diagnosis of yellow fever virus infection.

## Supporting information

S1 Table Details of kit evaluated. (PDF)

S2 Table Panel of samples used in evaluating the YF IgM test kit. (PDF)

S3 Table Contingency table for Standard Q Yellow Fever IgM RDT and YF IgM capture ELISA showing the basis for deriving formulas for calculating sensitivity, specificity, and positive and negative predictive values. (PDF)

S4 Table Table comparing results of the 21 positive YF IgM capture ELISA results with independent YF IgM test readings from three different biomedical scientists. RDT1, RDT2 and RDT3 represent independent visual reading and interpretation by three different biomedical scientists.
(PDF)

S5 Table Table comparing results of additional 60 positive YF IgM capture ELISA results with independent YF IgM test readings from three different biomedical scientists. (PDF)

S1 Text Brief description of Sandwich Enzyme Linked ImmunoSorbent Assay protocol for testing for the presence of anti-Yellow Fever specific IgM antibodies. Detailed description of the protocol can be found in the Manual for the monitoring of yellow fever virus infection. Geneva: World Health Organization; 2004. Pg 15;44 http://apps.who.int/iris/bitstream/10665/68715/1/WHO_IVB_04.08.pdf.
(PDF)
