# Supplementary material for: An evaluation of the diagnostic performance characteristics of the Yellow Fever IgM immunochromatographic rapid diagnostic test kit from SD Biosensor in Ghana
Source: PLoS One. 2022 Jan 7;17(1):e0262312. doi: 10.1371/journal.pone.0262312 (PMC8741057; doi:10.1371/journal.pone.0262312)
Supplement: S1 Table — (PDF) [file pone.0262312.s001.pdf]

Supplementary information S1 Table: Details of kit evaluated

**Product Name**	
**Manufacturer**	SD Biosensor Inc
**Catalog Number**	09YEL20D
**Batch Number**	QYE2018001-2
**Manufacturing Date**	2018.05.14
**Expiry Date**	2020.05.13
**Analyte**	IgM Antibodies
**Sample Type**	Serum, Plasma, Whole Blood
**Stated Accuracy**	None
