# Supplementary material for: An evaluation of the diagnostic performance characteristics of the Yellow Fever IgM immunochromatographic rapid diagnostic test kit from SD Biosensor in Ghana
Source: PLoS One. 2022 Jan 7;17(1):e0262312. doi: 10.1371/journal.pone.0262312 (PMC8741057; doi:10.1371/journal.pone.0262312)
Supplement: S2 Table — (PDF) [file pone.0262312.s002.pdf]

Supplementary information S2 Table: Panel of samples used in evaluating the YF IgM Test Kit

Group 1	21 archived samples that tested positive by YF IgM sandwich ELISA and confirmed by PRNT or RT-PCR.
Group 2	194 samples that tested negative by YF IgM sandwich ELISA but not tested by PRNT or RT-PCR irrespective of the presence of YF RNA.
Group 3	17 plasma samples collected from malaria positive children
Group 4	7 and 5 sera derived from hepatitis B and C positive cases respectively
Group 5	2 samples that tested positive for Dengue NS1 antigen
Group 6	60 samples from an ongoing outbreak that tested positive by YF IgM sandwich ELISA and confirmed by PRNT
