# Supplementary material for: An evaluation of the diagnostic performance characteristics of the Yellow Fever IgM immunochromatographic rapid diagnostic test kit from SD Biosensor in Ghana
Source: PLoS One. 2022 Jan 7;17(1):e0262312. doi: 10.1371/journal.pone.0262312 (PMC8741057; doi:10.1371/journal.pone.0262312)
Supplement: S3 Table — (PDF) [file pone.0262312.s003.pdf]

Supplementary Information S3 Table: Contingency Table for Standard Q Yellow Fever IgM RDT and YF IgM Capture ELISA showing the basis for deriving formulas for calculating sensitivity, specificity, and positive and negative predictive values

**Statistic**	**ELISA NEGATIVE**	**ELISA POSITIVE**	**TOTAL**
RDT NEGATIVE	TN	FN	TN+FN
RDT POSITIVE	FP	TP	FP+TP
TOTAL	TN+FP	FN+TP	**TOTAL**

TP= True Positive, FP =False Positive, FN=False Negative, TN= True Negative
