# Supplementary material for: An evaluation of the diagnostic performance characteristics of the Yellow Fever IgM immunochromatographic rapid diagnostic test kit from SD Biosensor in Ghana
Source: PLoS One. 2022 Jan 7;17(1):e0262312. doi: 10.1371/journal.pone.0262312 (PMC8741057; doi:10.1371/journal.pone.0262312)
Supplement: S4 Table — RDT1, RDT2 and RDT3 represent independent visual reading and interpretation by three different biomedical scientists. (PDF) [file pone.0262312.s004.pdf]

Supplementary information S4 Table: Table comparing results of the 21 positive YF IgM Capture ELISA results with independent YF IgM test readings from three different biomedical scientists

	IgM ELISA					Standard Q Yellow Fever IgM RDT			
Sample ID	Pt	Pt/N	2x C Ag	YF Ag	ELISA Result	RDT Result 1	RDT Result 2	RDT Result 3	RDT Final Result
In-House Positive Control	0.608	55.273	0.292	0.754	**Positive**	Positive	Positive	Positive	**Positive**
YF 311/16	0.295	4.92	0.444	0.517	**Positive**	Negative	Negative	Negative	**Negative**
YF 419/15	0.817	18.568	0.176	0.905	**Positive**	Positive	Positive	Positive	**Positive**
YF 418/15	1.185	32.92	0.164	1.267	**Positive**	Positive	Positive	Positive	**Positive**
YF 538/11	0.657	22.655	0.234	0.774	**Positive**	Positive	Positive	Positive	**Positive**
YF 557/11	0.849	29.276	0.238	0.968	**Positive**	Positive	Positive	Positive	**Positive**
YF 508/11	0.716	24.69	0.806	1.119	**Positive**	Positive	Positive	Positive	**Positive**
YF 512/11	0.524	18.069	0.396	0.722	**Positive**	Positive	Positive	Positive	**Positive**
YF 542/11	0.916	31.586	0.25	1.041	**Positive**	Positive	Positive	Positive	**Positive**
YF 602/11	0.98	33.793	0.244	1.102	**Positive**	Positive	Positive	Positive	**Positive**
YF 462/15	1.142	26.56	0.254	1.269	**Positive**	Positive	Positive	Positive	**Positive**
YF 432/15	1.276	29.674	1.19	1.871	**Positive**	Positive	Positive	Positive	**Positive**
YF 297/13	0.267	9.207	0.276	0.468	**Positive**	Positive	Positive	Positive	Positive
YF 412/13	0.275	5.29	0.194	0.372	**Positive**	Positive	Positive	Positive	**Positive**
YF 561/11	0.783	27	0.248	0.907	**Positive**	Positive	Positive	Positive	**Positive**
YF 560/11	0.767	26.448	0.258	0.896	**Positive**	Positive	Positive	Positive	**Positive**
YF 541/11	0.524	18.069	0.254	0.651	**Positive**	Positive	Positive	Positive	**Positive**
YF 42/11	0.9	31.034	0.248	1.024	**Positive**	Positive	Positive	Positive	**Positive**
YF 290/10	0.834	28.759	0.324	0.996	**Positive**	Positive	Positive	Positive	**Positive**
YF 192/12	0.523	2.08	0.37	0.707	**Positive**	Positive	Positive	Positive	**Positive**
YF 14/12	0.337	18.72	0.24	0.458	**Positive**	Positive	Positive	Positive	**Positive**
