# Supplementary material for: An evaluation of the diagnostic performance characteristics of the Yellow Fever IgM immunochromatographic rapid diagnostic test kit from SD Biosensor in Ghana
Source: PLoS One. 2022 Jan 7;17(1):e0262312. doi: 10.1371/journal.pone.0262312 (PMC8741057; doi:10.1371/journal.pone.0262312)
Supplement: S5 Table — (PDF) [file pone.0262312.s005.pdf]

Supplementary information S5 Table: Table comparing results of additional 60 positive YF IgM Capture ELISA results with independent YF IgM test readings from three different biomedical scientists

	YF IgM Capture ELISA					Standard Q YF IgM RDT			
**Sample ID**	**Avg OD VAg**	**P/N**	**Avg OD NAg**	**NBR**	**ELISA Result**	**RDT 1**	**RDT 2**	**RDT 3**	**RDT Final Result**
YF 1227/21	0.966	4.974	0.204	4.738	**Positive**	Positive	Positive	Positive	**Positive**
YF 1237/21	1.388	7.145	0.154	9.037	**Positive**	Positive	Positive	Positive	**Positive**
YF 1238/21	1.582	8.145	0.175	9.063	**Positive**	Positive	Positive	Positive	**Positive**
YF 1240/21	1.245	6.411	0.180	6.908	**Positive**	Positive	Positive	Positive	**Positive**
YF 1241/21	0.785	4.042	0.157	4.994	**Positive**	Positive	Positive	Positive	**Positive**
YF 1242/21	1.700	8.753	0.178	9.556	**Positive**	Positive	Positive	Positive	**Positive**
YF 1243/21	0.900	6.446	0.263	3.419	**Positive**	Positive	Positive	Positive	**Positive**
YF 1244/21	1.280	9.167	0.090	14.173	**Positive**	Positive	Positive	Positive	**Positive**
YF 1249/21	2.227	13.758	0.091	24.512	**Positive**	Positive	Positive	Positive	**Positive**
YF 1251/21	2.063	12.749	0.091	22.714	**Positive**	Positive	Positive	Positive	**Positive**
VHF/21/028	1.726	12.356	0.103	16.754	**Positive**	Positive	Positive	Positive	**Positive**
VHF/21/027	1.254	8.981	0.096	13.066	**Positive**	Positive	Positive	Positive	**Positive**
YF 1260/21	0.550	3.247	0.070	7.823	**Positive**	Positive	Positive	Positive	**Positive**
YF 1261/21	2.229	13.171	0.087	25.550	**Positive**	Positive	Positive	Positive	**Positive**
YF 1262/21	2.018	11.921	0.087	23.124	**Positive**	Positive	Positive	Positive	**Positive**
YF 1263/21	0.913	5.394	0.092	9.968	**Positive**	Positive	Positive	Positive	**Positive**
YF 1273/21	1.724	10.199	0.135	12.766	**Positive**	Positive	Positive	Positive	**Positive**
YF 1285/21	0.382	1.99	0.07	3.499	**Positive**	Positive	Positive	Positive	**Positive**
YF 1291/21	1.299	10.632	0.07	18.688	**Positive**	Positive	Positive	Positive	**Positive**
YF 1298/21	0.653	3.325	0.153	4.258	**Positive**	Negative	Negative	Negative	**Negative**
YF 1315/21	2.09	10.576	0.191	10.926	**Positive**	Positive	Positive	Positive	**Positive**
YF 1316/21	1.743	8.821	0.16	10.922	**Positive**	Positive	Positive	Positive	**Positive**
YF 1317/21	1.823	9.228	0.222	8.204	**Positive**	Positive	Positive	Positive	**Positive**
YF 1322/21	0.607	3.663	0.178	3.409	**Positive**	Positive	Positive	Positive	**Positive**
YF 1324/21	1.681	10.144	0.331	5.073	**Positive**	Positive	Positive	Positive	**Positive**
YF 1342/21	1.867	9.349	0.173	10.768	**Positive**	Positive	Positive	Positive	**Positive**
YF 1360/21	0.796	4.021	0.136	5.852	**Positive**	Positive	Positive	Positive	**Positive**
YF 1362/21	2.548	13.063	0.091	27.979	**Positive**	Positive	Positive	Positive	**Positive**
YF 1363/21	0.257	1.320	0.085	3.037	**Positive**	Negative	Negative	Negative	**Negative**
YF 1366/21	2.424	12.428	0.092	26.234	**Positive**	Positive	Positive	Positive	**Positive**
YF 1370/21	2.128	11.125	0.078	27.306	**Positive**	Positive	Positive	Positive	**Positive**
YF 1400/21	0.749	8.302	0.059	12.717	**Positive**	Positive	Positive	Positive	**Positive**
YF 1401/21	1.460	16.173	0.062	23.573	**Positive**	Positive	Positive	Positive	**Positive**
YF 1405/21	1.568	13.326	0.095	16.448	**Positive**	Positive	Positive	Positive	**Positive**

YF 1407/21	1.674	14.224	0.097	17.254	**Positive**	Positive	Positive	Positive	**Positive**
YF 1408/21	1.201	10.207	0.098	12.214	**Positive**	Positive	Positive	Positive	**Positive**
YF 1409/21	1.524	12.955	0.100	15.294	**Positive**	Positive	Positive	Positive	**Positive**
YF 1411/21	1.353	11.101	0.108	12.543	**Positive**	Positive	Positive	Positive	**Positive**
YF 1418/21	1.508	8.530	0.128	11.741	**Positive**	Positive	Positive	Positive	**Positive**
YF 1425/21	0.709	3.987	0.251	2.827	**Positive**	Positive	Positive	Positive	**Positive**
YF 1426/21	2.469	13.893	0.202	12.220	**Positive**	Positive	Positive	Positive	**Positive**
YF 1427/21	2.341	13.173	0.252	9.288	**Positive**	Positive	Positive	Positive	**Positive**
YF 1431/21	1.775	9.717	0.228	7.774	**Positive**	Positive	Positive	Positive	**Positive**
YF 1433/21	1.204	6.593	0.216	5.576	**Positive**	Positive	Positive	Positive	**Positive**
YF1 440/21	2.096	11.998	0.180	11.643	**Positive**	Positive	Positive	Positive	**Positive**
YF 1445/21	2.421	13.722	0.207	11.671	**Positive**	Positive	Positive	Positive	**Positive**
YF 1446/21	2.746	15.564	0.285	9.631	**Positive**	Positive	Positive	Positive	**Positive**
YF 1448/21	1.408	7.980	0.236	5.955	**Positive**	Positive	Positive	Positive	**Positive**
YF 1452/21	1.481	9.895	0.102	14.520	**Positive**	Positive	Positive	Positive	**Positive**
YF 1455/21	1.415	9.457	0.106	13.352	**Positive**	Positive	Positive	Positive	**Positive**
YF 1457/21	1.404	9.379	0.111	12.684	**Positive**	Positive	Positive	Positive	**Positive**
YF 1460/21	2.046	11.133	0.144	14.166	**Positive**	Positive	Positive	Positive	**Positive**
YF 1464/21	1.678	9.131	0.137	12.209	**Positive**	Positive	Positive	Positive	**Positive**
YF 1465/21	1.850	10.068	0.118	15.624	**Positive**	Positive	Positive	Positive	**Positive**
YF 1466/21	2.264	12.679	0.170	13.300	**Positive**	Positive	Positive	Positive	**Positive**
YF 1467/21	1.912	10.706	0.129	14.869	**Positive**	Positive	Positive	Positive	**Positive**
YF 1470/21	1.429	8.001	0.097	14.795	**Positive**	Positive	Positive	Positive	**Positive**
YF 1471/21	1.739	9.737	0.124	13.995	**Positive**	Positive	Positive	Positive	**Positive**
YF 1474/21	1.486	8.406	0.163	9.092	**Positive**	Positive	Positive	Positive	**Positive**
YF 1476/21	2.158	12.212	0.198	10.878	**Positive**	Positive	Positive	Positive	**Positive**
