# Supplementary material for: An evaluation of the diagnostic performance characteristics of the Yellow Fever IgM immunochromatographic rapid diagnostic test kit from SD Biosensor in Ghana
Source: PLoS One. 2022 Jan 7;17(1):e0262312. doi: 10.1371/journal.pone.0262312 (PMC8741057; doi:10.1371/journal.pone.0262312)
Supplement: S1 Text — Detailed description of the protocol can be found in the Manual for the monitoring of yellow fever virus infection. Geneva: World Health Organization; 2004. Pg 15;44 http://apps.who.int/iris/bitstream/10665/68715/1/WHO_IVB_04.08.pdf. (PDF) [file pone.0262312.s006.pdf]

Supplementary information S1 Text: Brief Description of Sandwich Enzyme Linked ImmunoSorbent Assay Protocol for Testing for the Presence of anti-Yellow Fever Specific IgM Antibodies

- Micro-wells were coated with 10 µl of stock goat anti human IgM (Kirkegaard and Perry Laboratories) and incubated at 4 ^{o}C overnight.
- The micro-well plate was then washed using PBS-Tween 20 (Sigma-Aldrich Co.), blocked with 10% foetal bovine serum in PBS/Tween milk and incubated for 1 hour at 37^{0}C in a water bath.
- Test serum samples, positive and negative control sera were added after washing the wells with PBS-Tween 20.
- After a series of incubation and washing steps, substrate TMB was added and the reaction was stopped by the addition of 2M H_2SO_4. The optical densities of the samples were read at 450nm using a spectrophotometer.

All presumptive ELISA positive samples were confirmed by PRNT and or RT-PCR at the WHO regional laboratory in Dakar, Senegal.
